# Correspondence

**Published:** 1881-10

**Authors:** H. R. Hopkins

**Affiliations:** Buffalo


### Correspondence.

*Messrs. Editors:*—In the number of your journal for August, 1871, appeared a letter of mine upon the subject of "Homœopathy and Regular Medicine," in which views were advanced not in accordance with those generally esteemed orthodox. The drift of my communication is indicated by the following quotation:

"In view of these facts, what should the regular profession do in the matter? Shall we continue to call ourselves 'the profession,' and neither by public act or private word allow that there is any other? Shall we continue a line of treatment condemned by law and by experience, treatment which only makes homœopathy notorious and ourselves disgraceful; or shall we submit gracefully to the laws of the State and public opinion, and proffer to the homœopathic profession those amenities which should exist between professional equals? Invite them to their rights in our county medical societies; when called by their patrons, attend with them in consultation; when wished by our patients, ask them to attend in consultation with us. If they have any superior knowledge in the management of disease, or the protection of health, our duty to our patrons requires us to avail ourselves of that knowledge. If we possess the greater professional ability, they and their patrons will find it out. If we hold back from this, we may reasonably be charged with having little confidence in our doctrines. If we go into it, I rest my faith upon the survival of the fittest."

The reception of these views by the profession was, to say the least, not cordial. The men who found themselves called upon to make reply had, as usual, the option of answering the arguments or of abusing the writer, and because the task was easier, or more congenial, chose to do the latter.

Accordingly the next issue of the JOURNAL contained a letter selected from many others as a rejoinder, which made no allusion to the matter under discussion, but which was well filled with personal abuse of myself. Also an editorial containing the following reference to my letter:

"If our readers and correspondents will forgive us for publishing the paper for professional reading, and for suppressing their very complete and very satisfactory rejoinders, we will regard the unpardonable sins of editorial life blotted out and solemnly promise with penitence and prayer to never again thus trespass upon their indulgence."

Inasmuch as the letter was written under the impression that I had something to say to the profession, and having no desire for personal controversy, the above went unnoticed. This was ten years ago this month.

In the Boston *Medical and Surgical Journal* of August 25, 1881, there appears, reprinted from advance sheets, an address on Medicine before the British Medical Association at its late meeting, by John Syer Bristowe, M. D., upon the subject of "Homœopathy," of which the following is the conclusion:

"But it may be replied, if those men are honest and educated, and at the same time duly qualified practitioners in medicine, how can they believe and how can they practice such a palpable imposition as homœopathy? Well, gentlemen, it is very difficult to account for the beliefs and vagaries of the human intellect. It is only occasionally that our convictions are the result of conscious reasoning. For the most part they arise in the mind, and take possession of it, we know not how or why; and our reasonings in regard to them (if we reason at all) are merely special pleadings prompted by the very convictions they seem to us to determine—in other words, they are not the foundations of our beliefs at all, but exhalations from them. It is not surprising, therefore, that, even on matters of supreme importance, irreconcilable differences of opinion prevail, aye, even amongst men of high integrity and cultivated intellect. And if we desire to live broad and unselfish lives, we must be slow to condemn all those who entertain convictions which to us seem foolish or mischievous, and logically untenable, or to refuse to co-operate with them. * * *

"I ask you, gentlemen, to forbear with me if I push my argument to the logical conclusion, and venture now to express an opinion which is opposed to the opinion which many, perhaps most, of you entertain. I do not ask you to agree with it; still less do I ask you to accept it. But I ask you to consider it; and I am content to believe that, if it be just, it will ultimately prevail. It is that, when homœopathists are honest, and well-informed, and legally qualified practitioners of medicine, they should be dealt with as if they were honest and well-informed and qualified. I shall not discuss the question whether we can, with propriety or with benefit to our patients, meet homœopaths in consultation. I could, however, I think, adduce strong reasons in favor of the morality of acting thus, and for the belief that good to the patient would generally ensue under such circumstances. I shall not consider at length whether the dignity of the profession would be compromised by habitual dealing with homœopathists. But I may observe that it is more conducive to the maintenance of true dignity to treat with respect and consideration, and as if they were honest, those whose opinions differ from ours, than to make broad our phylacteries and enlarge the borders of our garments, and wrap ourselves up, in regard to them, in Pharisaic pride. I appeal, gentlemen, in support of my contention, to other considerations. It has been held that to break down the barriers that at present separate us from homœopathists would be to allow the poison of quackery to leaven the mass of orthodox medicine. But who that has any trust in his profession, any scientific instinct, any faith in the ultimate triumph of truth, can entertain any such fear? All the best physicians of old times, all the greatest names in medicine of the present day, are with us; and we know that as a body we are honest seekers after truth. What have we to fear from homæopathy? Bigots are made martyrs by persecution; false sects acquire form and momentum and importance mainly through the opposition they provoke. When persecution ceases would-be martyrs sink into insignificance; in the abscence of the stimulus of active opposition sects tend to undergo disintegration and to disappear. The rise and spread of homœopathy have been largely due to the strong antagonism it has evoked from the schools of orthodox medicine, and to the isolation which has thus been imposed on its disciples. If false, as we believe it to be, its doom will be sealed when active antagonism and enforced isolation no longer raise it into fictitious importance. At any rate, breadth of view, and liberality of conduct, are the fitting characteristics of men of science."

I have sent you this extract because of its calm judicial thought upon a subject of great, natural difficulties, and one upon which we can seldom think at all without the bias of personal feeling; also because the eminent writer considered the theme worthy the attention of the British Medical Association; and finally, because his views upon the question are quite similar to those I ventured to express ten years ago.

H. R. Hopkins, M. D.

Buffalo, September 5, 1881.

